# Deubiquitinating enzyme USP41 promotes lung cancer cell proliferation and migration

**DOI:** 10.1111/1759-7714.13843

**Published:** 2021-02-22

**Authors:** Jiaqi Ji, Shuping Yang, Lingling Zu, Yongwen Li, Ying Li

**Affiliations:** ^1^ Department of Pulmonary and Critical Care Medicine Sichuan Academy of Medical Sciences & Sichuan Province People's Hospital Chengdu China; ^2^ Department of Anesthesiology Huashan Hospital Affiliated to Fudan University Shanghai China; ^3^ Key Laboratory of Lung Cancer Metastasis and Tumor Microenvironment, Tianjin Lung Cancer Institute Tianjin Medical University General Hospital Tianjin China; ^4^ Key Laboratory of Post‐Neuroinjury Neuro‐repair and Regeneration in Central Nervous System, Ministry of Education, Tianjin Neurological Institute Tianjin Medical University General Hospital Tianjin China

**Keywords:** deubiquitylating enzyme, invasion, migration, non‐small cell lung cancer, USP41

## Abstract

**Background:**

To reveal the function of deubiquitylating enzyme USP41 in lung adenocarcinoma.

**Methods:**

The relationship between USP41 and lung cancer was determined by analyzing data from The Cancer Genome Atlas (TCGA). A549 and H1299 cell lines were transfected with short hairpin RNA against USP41 (shUSP41 group) or negative control (shCon group). Western blotting was used to verify the transfection efficacy and marker expression. Cell proliferation and apoptosis were analyzed by EdU assay, MTT assay, and flow cytometry after USP41 knockdown. Transwell assay was used to determine the effect of USP41 downregulation on cell migration.

**Results:**

Analysis of lung cancer data from TCGA database indicated a higher level of USP41 expression in lung cancer tumor tissue compared with that in noncancerous tissue, and USP41 overexpression was correlated with poor overall survival of lung cancer patients (*p* < 0.01). The outcomes of the EdU, MTT, and flow cytometry assays indicated decreased cell proliferation and enhanced apoptosis in shUSP41‐transfected cells. Transwell assay further demonstrated that USP41 knockdown increased the migration rate of A549 and H1299 cells.

**Conclusions:**

In our study, USP41 was overexpressed in lung cancer tissue and associated with poor prognosis of lung cancer. USP41 knockdown inhibits cell proliferation and migration and induces cell apoptosis of lung cancer.

## Graphical abstract

USP41 is overexpressed in lung cancer tissue and associated with poor prognosis of lung cancer. USP41 overexpression promotes cell proliferation and migration and inhibits apoptosis of lung cancer cells.

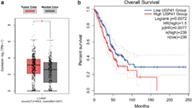

## INTRODUCTION

Lung cancer has recently become the leading cause of cancer deaths worldwide, among which non‐small cell lung cancer (NSCLC) has shown a significant growth trend as its main pathological type.^1^, ^2^ Numerous studies have reported that the functions of the ubiquitin family (deubiquitylating enzymes, DUBs) are closely related to the occurrence and development of NSCLC.^3^ The ubiquitin‐specific protease (USP) subfamily has the most members, and it is also the most widely studied family.^4^ Members of the USP family are structurally diverse cysteine proteases that remove the ubiquitin chain from the target protein by hydrolysis, promote protein stability and function, and are involved in cellular DNA repair, cell cycle progression, and gene transcriptional regulation.^5^, ^6^, ^7^ Because the number of studies targeting specifically activated members of the USP family for drug development is increasing, it is crucial to elucidate the function and mechanism of action of members of the USP family. The role of USP41, a member of the USP family, in NSCLC is unclear. Therefore, the aim of this study was to analyze the expression and function of USP41 in lung cancer to provide more evidence for its use as a target in the clinical treatment of lung cancer.

## METHODS

### Online database analysis

The GEPIA website (http://gepia2.cancer-pku.cn/#help) is an online database that includes information about cancer patients in The Cancer Genome Atlas (TCGA) database and gene expression data of normal human tissue in the Genotype‐Tissue Expression (GTEx) database. We used this website to collectively analyze data from 483 lung cancer patients in the TCGA database and the normal tissue data in the GTEx database. We compared the differences in USP41 expression in lung cancer and normal tissue and analyzed the overall survival rate of lung cancer patients using the Kaplan–Meier method.

### Cell lines

We purchased NSCLC cell lines A549 and H1299 from the Shanghai Cell Bank of the Chinese Academy of Sciences (China). The two cell lines were cultured in Roswell Park Memorial Institute (RPMI) 1640 medium containing 10% fetal bovine serum and placed in a constant temperature incubator at 37°C with 5% CO_2_ and humidity saturation. The experiment was performed on cells in the logarithmic growth stage.

### Antibodies and reagents

RPMI 1640 cell culture medium, fetal bovine serum, penicilli streptomycin mixture, trypsin, RIPA protein lysate, and 3‐(4,5‐dimethyl‐2‐thiazolyl)‐2,5‐diphenyltetrazolium bromide (MTT) reagent were purchased from Sigma‐Aldrich Corporation. USP41 antibody was purchased from Thermofisher Inc. and GAPDH antibody was purchased from Cell Signaling Technology Inc. Kits for bicinchoninic acid (BCA) protein quantitation, enhanced chemiluminescence (ECL), EdU staining, and apoptosis were purchased from Thermo Fisher. Transwell migration chambers were purchased from Corning Inc.

### Construction of short hairpin RNA against USP41 (shUSP41) vector and cell transfection

The shUSP41 interference sequences were as follows: shUSP41‐1: sense, 5′‐CACCGCGTCTTGTTCAGGGCTCATCTCAAGAGGATGAGCCCTGAACAAGACGC‐3′ and antisense, 5′–AAAAGCGTCTTGTTCAGGGCTCATCCTCTTGAGATGAGCCCTGAACAAGACGC‐3′; and ShUSP41‐2: sense, 5′‐CACCGGGCTCATCAGTGTCAGTACGTCAAGAGCGTACTGACACTGATGAGCCC‐3′ and antisense, 5′‐AAAAGGGCTCATCAGTGTCAGTACGCTCTTGACGTACTGACACTGATGAGCCC‐3′. The interference sequence was connected with a pLKO vector to obtain pLKO‐shUSP41 core vector, which was then cotransfected with a pVSVG, pREV, and pGAG packaging system into HEK293T cells. After 24 h, we collected the supernatant containing the viral particles by centrifugation, filtered it through a 0.45 μ filter, and determined the viral titer by the dilution method to obtain the appropriate concentration of infected cells. Then, A549 and H1299 cells were inoculated into a 24‐well plate at a density of 3 × 10^4^ cells/well, and virus and polybrene were added to each well. After 48 h, the medium containing puromycin was changed, and the plates were cultured for one week. The cell lines that were stably transfected with shUSP41 were screened out, and the successful knockout of USP41 was determined by western blot assay.

### Western blotting

The transfected NSCLC cells were lysed, and the total protein was extracted with RIPA buffer and quantified using a BCA kit. Following denaturation in the sample buffer at 100°C for 5 min, 30 μg of each protein sample was subjected to 10% sodium dodecyl sulfate‐polyacrylamide gel electrophoresis for 50 min. Sodium dodecyl sulfate‐polyacrylamide gel was placed on the nitrocellulose membrane, and protein transfer was performed at a constant current of 200 mA for 60 min. After blocking the nitrocellulose membrane in 5% skim milk at room temperature for 2 h, it was incubated at 4°C overnight in a proportional dilution of primary protein antibody. The next day, the membrane was washed three times with phosphate‐buffered saline (PBS) with Tween 20 (PBST) solution, placed in the secondary antibody, incubated at room temperature for 45 min, and washed with PBST. Finally, ECL solution was used to incubate and develop the color of the membrane to capture the target band.

### EdU assay

The target cells were seeded in 96‐well plates and cultured for 48 h with 3–5 wells in each group. Then, 100 μl diluted EdU solution (final concentration, 50 M EdU) was added to each well and incubated for 2 h. The cells were fixed with 50 μl of 4% paraformaldehyde for 30 min, incubated with 100 μl permeation agent (containing 5% Triton X‐100 in PBS) for 15 min, and washed twice with PBS between each step. Thereafter, 100 μl diluted reaction solution was added to each well for staining and incubated on a shaker for 20 min before discarding the solution. Next, 100 μl permeation agent was added for 10 min. Finally, 200 μl 4′,6‐diamidino‐2‐phenylindole staining solution was added to each well and incubated in the dark for 5 min, followed by three washes with PBS. The number of positive cells was recorded, and the proliferation rate was calculated.

### MTT assay

The USP41‐knockout A549 and H1299 cells and the control cells were digested and inoculated into 96‐well plates with 3000 cells per well and 100 μl fresh medium, which was changed every 24 h. At 24, 48, 72, and 96 h, 20 μl of MTT solution (5 mg/L) was added to each well, respectively. After 4 h, the medium was removed from the 96‐well plate, and 150 μl DMSO was added to each well. The absorbance value of each group at 490 nm was determined by a microplate reader and used for subsequent statistical analysis.

### Migration assay

The cells were resuspended in serum‐free medium to form a single cell suspension, and 3 × 10^4^ cells/well were seeded in the upper compartment of the Transwell chamber. A complete medium containing 10% fetal bovine serum was added to the lower compartment, and the chamber was placed in an 37°C incubator with 5% CO2 and humidity saturation. After 24 h, the cells that could not pass through the upper layer of the chamber were wiped off with a cotton swab, and the cells were stained with 0.1% crystal violet at room temperature for 15 min. We randomly selected five fields from each chamber for photography, and ImageJ software (https://imagej.nih.gov/ij/download.html) was used for quantitative analysis.

### Flow cytometry assay

The cells were washed twice with PBS and centrifuged to remove the supernatant. Then, they were incubated in diluted annexin V staining solution for 30 min in each tube at room temperature, followed by the addition of 1 μl propidium iodide (PI) staining solution to each tube and incubation at room temperature for 5 min. Apoptosis was detected using a FACS Calibur flow cytometer (BD Biosciences, USA) for subsequent analysis and representation.

### Statistical analysis

SPSS 21.0 (IBM Corp., USA) and GraphPad Prism 7.0 (GraphPad Software, USA) were used for statistical analysis and data mapping. The data with a normal distribution were expressed as means ± SD. Student's *t*‐test or one‐way ANOVA was used to compare the data from the two independent samples. A *p*‐value <0.05 was considered statistically significant.

## RESULTS

### Correlation analysis between USP41 and lung cancer prognosis

**FIGURE 1:**
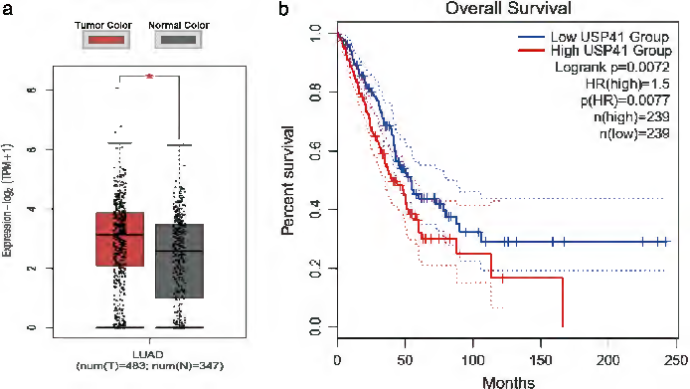
USP41 expression in lung cancer patients and its effect on prognosis. (a) Analysis of TCGA and GTEx databases showed that the level of deubiquitination enzyme USP41 in lung cancer patients was significantly higher than that in normal tissue. (b) The overall survival rate of lung cancer patients with a high level of USP41 expression was poor

Using the GEPIA website, we first analyzed the expression of USP41 in lung cancer patients in TCGA database and in normal human tissue in the GTEx database. The level of deubiquitinase USP41 in lung cancer patients was significantly higher than that in normal tissue (Figure 1(a)), and a high expression level of USP41 predicted a poor overall survival rate in lung cancer patients (Figure 1(b)), indicating that USP41 promotes lung cancer progression and may be a prognostic indicator for lung cancer patients.

### USP41 affects the proliferation of lung cancer cells

**FIGURE 2:**
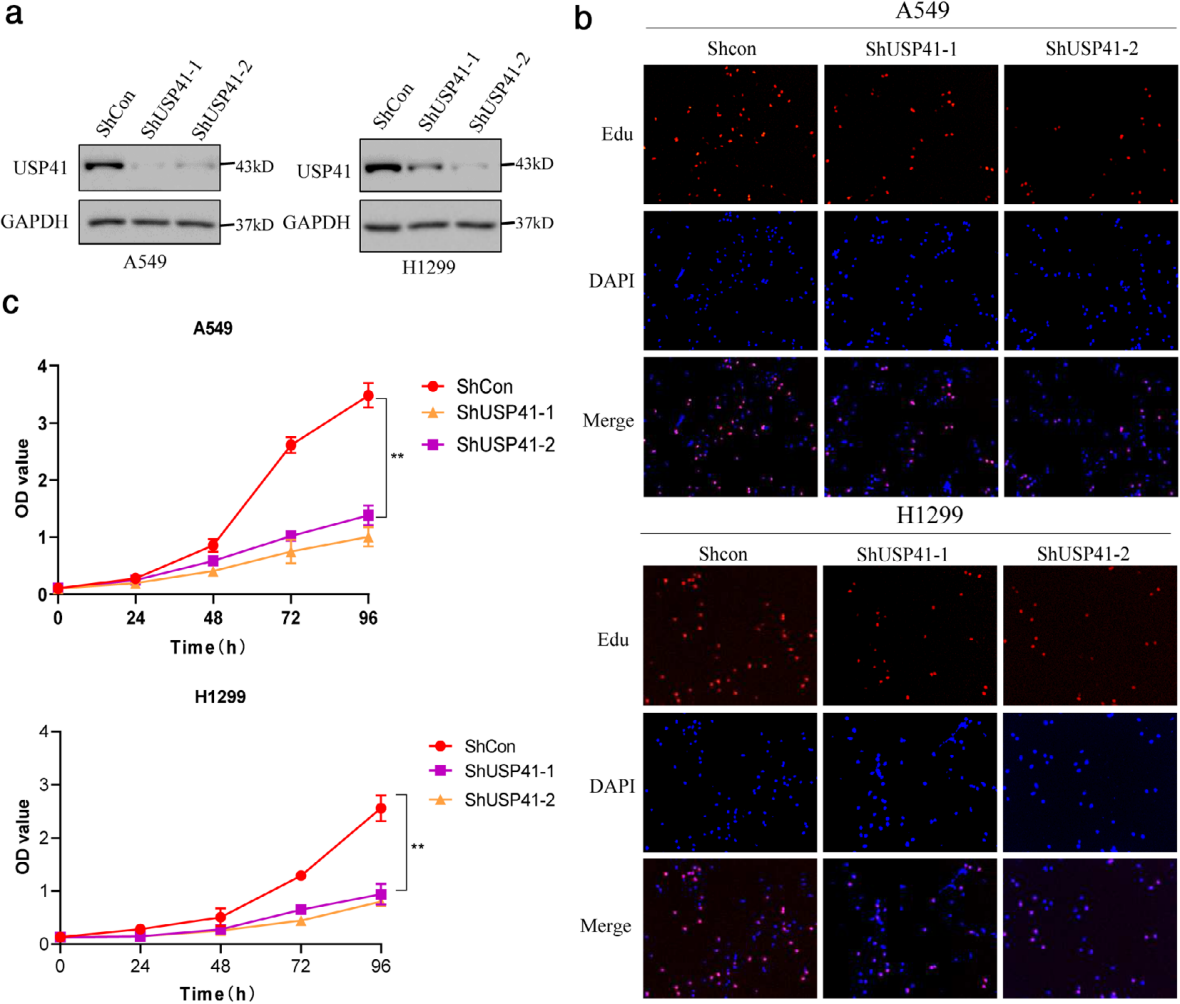
USP41 affects lung cancer cell proliferation. Three independent experiments were performed. **p < 0.01 (a) Western blot assay confirmed that USP41 knockdown was effective in A549 and H1299 lung cancer cell lines. (b) The EdU assay showed that the proliferation ability of A549 and H1299 cells was significantly decreased after USP41 knockdown compared with the control group. (c) MTT assay showed that the survival rate of A549 and H1299 cells cultured for 96 h after USP41 knockdown was significantly lower than that of the control group

We investigated whether USP41 affects the proliferation of lung cancer cells to further determine the oncogenic role of USP41 in lung cancer. USP41 knockdown was performed on A549 and H1299 lung cancer cell lines using shRNA, and the transfection efficiency was evaluated by western blot assay to determine the effectiveness of shUSP41 (Figure 2(a)). We analyzed the proliferation and survival of lung cancer cells using EdU and MTT assays. A549 and H1299 cells with USP41 knockdown were significantly less proliferative and the cell survival rate after 96 h of culture was significantly lower compared with those cells transfected with the control plasmid (Figure 2(b) and (c)).

### USP41 knockdown promotes lung cancer cell apoptosis

**FIGURE 3:**
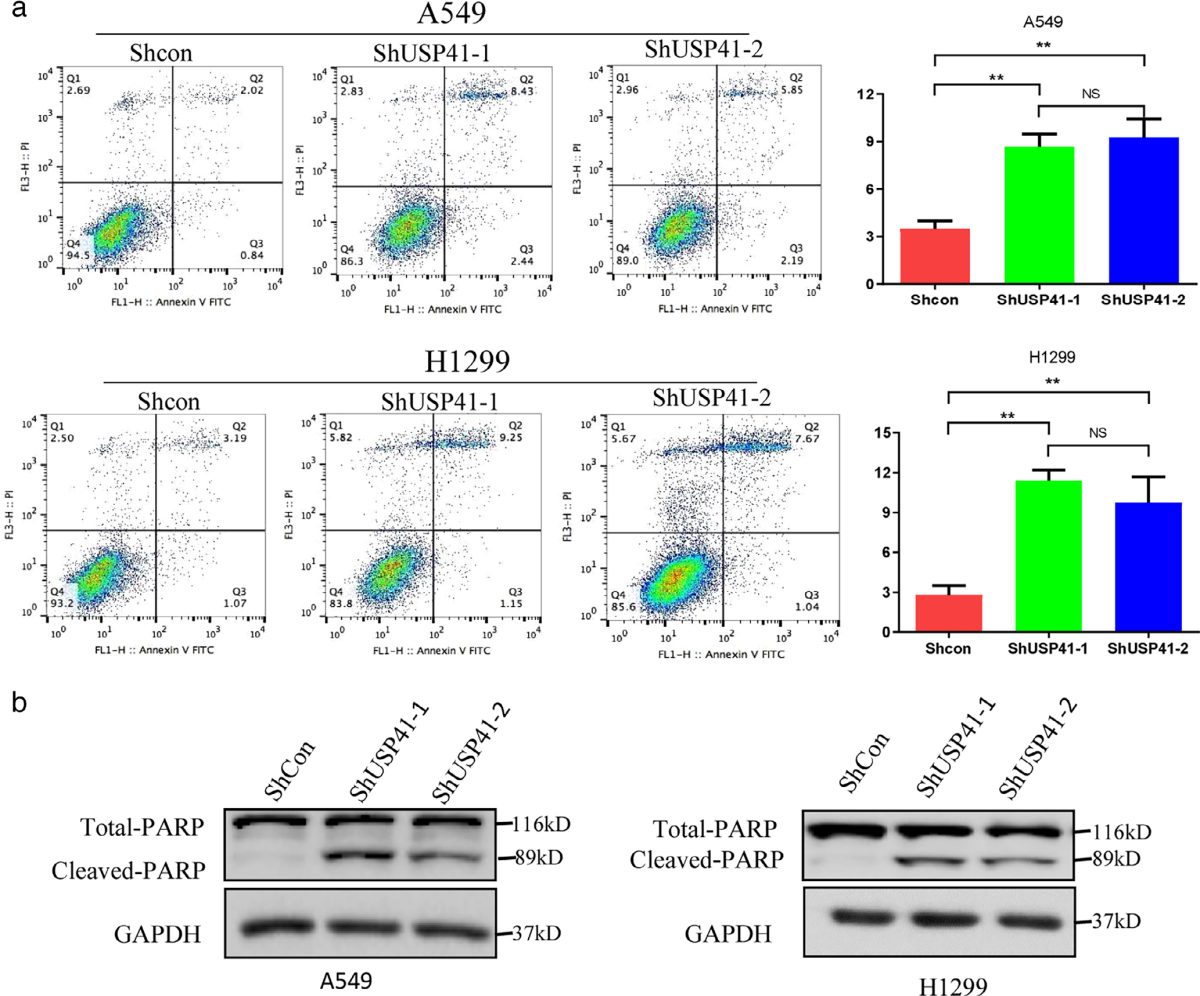
Effect of USP41 knockdown on lung cancer cell apoptosis. Three independent experiments were performed. **p < 0.01. (a, b) The amount of apoptotic A549 and H1299 cells increased when compared to the control group. (b) USP41 knockdown significantly increased cleaved PARP proteins in A549 and H1299 cells

We used annexin V/PI double‐staining to detect apoptosis in lung cancer cells. The apoptosis levels of A549 and H1299 lung cancer cells were significantly increased after USP41 knockdown. Compared with the control group, apoptosis of A549 cells increased from 2% to 8% (Figure 3(a)), and apoptosis of H1299 cells increased from 3% to approximately 10% (Figure 3(b)). Western blot results showed that USP41 knockdown significantly increased apoptotic marker proteins in A549 and H1299 cells (Figure 3(c)).

### Effect of USP41 knockdown on lung cancer cell migration

**FIGURE 4:**
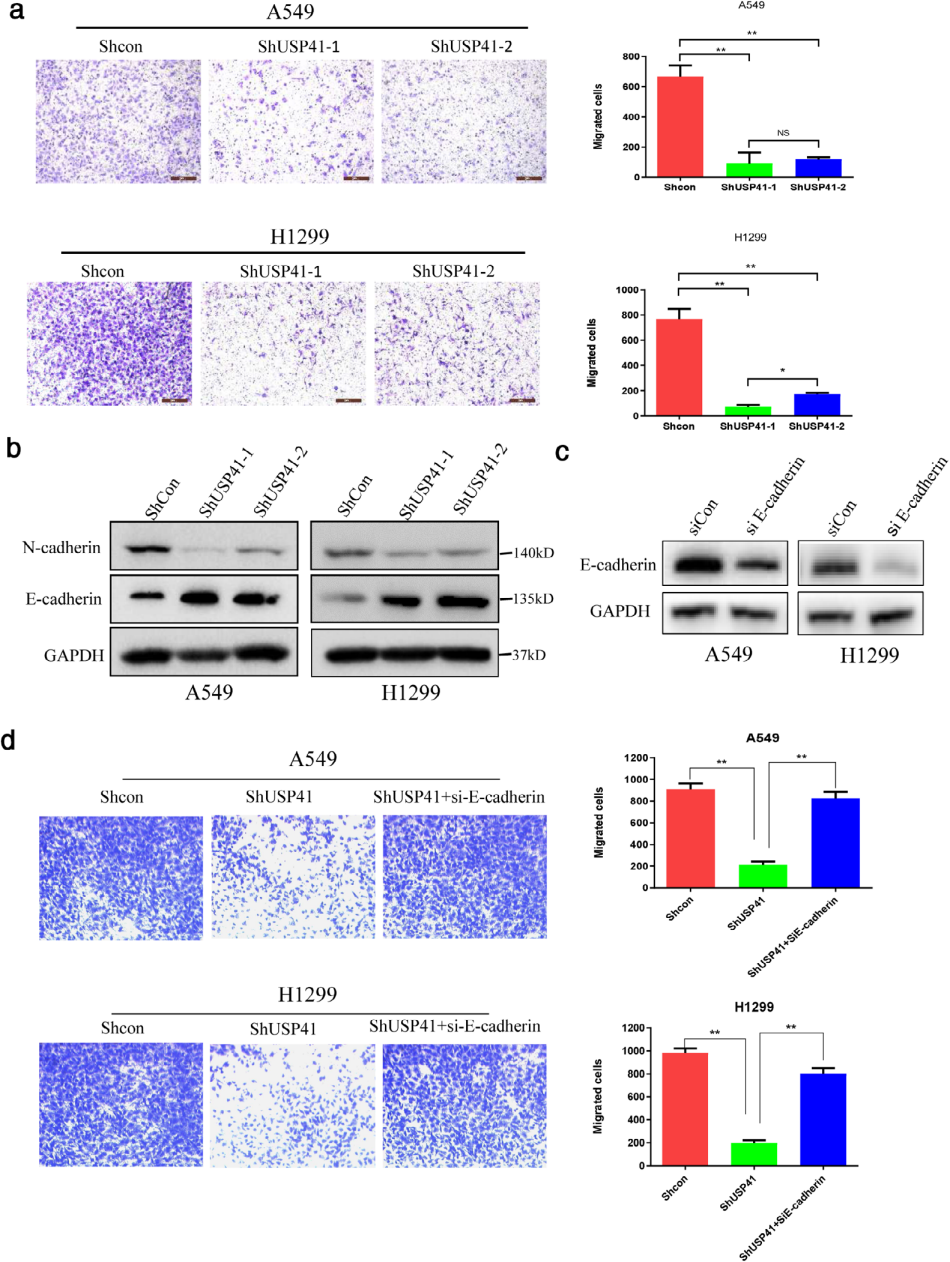
Effect of USP41 knockdown on lung cancer cell migration. Three independent experiments were performed. **p < 0.01. (a, b) The cell migration of A549 and H1299 cells were significantly reduced when compared to the control group. (c) Protein expression of N‐cadherin and E‐cadherin in the control and USP41‐knockdown group detected by western blot

Transwell assay results showed that the number of migratory A549 and H1299 cells significantly decreased compared with the control group after USP41 knockdown (Figure 4(a)). Western blot results showed that after USP41 knockdown, the expression of N‐cadherin in A549 and H1299 cells was significantly decreased, while the expression of E‐cadherin, a migration inhibitor, was significantly increased (Figure 4(b)). These results indicated that USP41 promoted lung cancer cell migration. We knocked down E‐cadherin in lung cancer cell lines using siRNA (Figure 4(c)), and demonstrated that E‐Cadherin knockout offseted the effect of inhibition of USP41 on cell migration, and inhibition of USP41 may reverse EMT of lung cancer cells (Figure 4(d)).

## DISCUSSION

Numerous clinical studies have shown that elevated tumor maker levels are a major feature of cancer occurrence. Ubiquitination and deubiquitination are important methods that regulate protein degradation and have attracted much attention in the field of cancer research.^8^ The protein is linked to the ubiquitin chain under the action of a ubiquitin enzyme and then recognized by the proteasome and hydrolyzed. The DUBs are capable of regulating protein homeostasis and function by removing ubiquitin from proteins and participating in critical cellular physiological processes, such as cell signaling, DNA repair, cell cycle progression, and apoptosis.^1^, ^9^ When deubiquitinase is abnormally activated, the proteolytic process of corresponding oncoprotein substrates is impaired to varying degrees,^10^ resulting in increased protein stability and the continuous function of oncoproteins, which promotes tumor formation and progression.

The USP family is the largest deubiquitination enzyme subfamily, comprising over 50 members. USP structure is highly conserved, and USPs participates in many physiological processes, such as DNA repair, cell cycle progression, and transcriptional regulation.^7^ USP family members play different functions in cancer, and most of them, such as USP1, USP2, USP7, USP14, and USP17, contribute to cancer promotion. USP2 expression levels in breast cancer, cervical cancer, glioma, and prostate cancer tissue are significantly higher than those in the corresponding paracancer tissue.^11^, ^12^, ^13^, ^14^ USP7 is highly expressed in lung cancer and multiple myeloma^15^, ^16^ and is associated with poor prognosis of colon cancer, glioma, liver cancer, and other cancers.^17^, ^18^, ^19^ A few USPs, such as CYLD, are characterized as tumor suppressor factors that inhibit the occurrence or metastasis of liver, skin, and colon cancers.^20^, ^21^, ^22^ Also, several USPs play both carcinogenic and anticancer roles in different cancers. For example, USP9X inhibits tumor formation in colon cancer, while it is highly expressed in lung cancer and various blood cancers.^23^, ^24^, ^25^

NSCLC studies have shown that high expression levels of USP5 and USP7 in lung cancer tissue promote lung cancer cell proliferation by stabilizing beta‐catenin.^15^, ^26^, ^27^ USP14 expression by miR‐124 promotes stem cell characteristics of NSCLC cells and gemcitabine resistance,^28^ and USP17 expression is associated with NSCLC metastasis and poor prognosis.^29^, ^30^ Furthermore, CYLD is a tumor suppressor, and its defects lead to lung cancer metastasis.^31^

At present, the function and regulatory mechanism of many USP family members, including USP41, have not been studied. In this study, the analysis of lung cancer cases in TCGA database showed that USP41 was highly expressed in lung cancer tumor tissue and predicted poor overall survival rate of lung cancer patients, indicating that USP41 functions as an oncogene in lung cancer. MTT, EdU, flow cytometry, and transwell assays further determined that USP41 enhanced the proliferation and migration of lung cancer cells. Based on the mechanisms of involvement of the USP family in many solid tumors,^14^, ^32^, ^33^ we can speculate that USP41 acts through regulating MDM2, Cyclin D1, FAS, and TGFBR1, which promote tumor cell survival and proliferation, inhibit cell apoptosis, promote cell migration, and may be associated with the beta‐catenin signaling pathway. We intend to continue our exploration of USP41‐specific regulatory mechanisms in NSCLC in subsequent experiments. Due to the important role of the USP family in cancer, many USP inhibitors have been developed. For example, small‐molecule inhibitor P5091 inhibits USP7 in colon cancer and chronic leukemia,^16^, ^17^, ^34^ and small‐molecule inhibitor b‐AP15 plays a significant role in inhibiting the cell growth of leukemia and esophageal squamous cell carcinoma.^35^, ^36^ Further studies on USP41 in NSCLC may provide more evidence for its use as a target in the clinical treatment of lung cancer.

## CONFLICT OF INTEREST

The authors have no conflicts of interest to declare.
